# A Novel Cell Culture System to Improve MRGPRX2 Research in Human Skin Mast Cells

**DOI:** 10.1002/clt2.70112

**Published:** 2025-10-16

**Authors:** Zhuoran Li, Jean Schneikert, Anja Wegner, Torsten Zuberbier, Magda Babina

**Affiliations:** ^1^ Institute of Allergology Charité—Universitätsmedizin Berlin Corporate Member of Freie Universität Berlin and Humboldt Universität zu Berlin Berlin Germany; ^2^ Fraunhofer Institute for Translational Medicine and Pharmacology ITMP Immunology and Allergology Berlin Germany; ^3^ Department of Dermatology The Second Hospital of Tianjin Medical University Tianjin China

**Keywords:** degranulation, mast cells, media, MRGPRX2, skin, tryptase

## Abstract

**Background:**

MRGPRX2, a receptor central to mast cell (MC) activation and related skin diseases, is selectively expressed in skin MCs but downregulated during culture by stem cell factor (SCF) and interleukin‐4 (IL‐4).

**Objective:**

To identify culture conditions that preserve MC viability while restoring MRGPRX2 expression and function.

**Methods:**

Human skin MCs were cultured in standard or serum‐free Accell medium (both with SCF). After 3 days, MRGPRX2 expression was assessed by flow cytometry, degranulation by mediator release assays, and signaling by Western blotting.

**Results:**

Accell medium increased MRGPRX2 expression to ~1.7‐fold and enhanced degranulation to Substance P and codeine. It also promoted stronger and more sustained ERK and AKT phosphorylation, while FcεRI‐mediated responses were largely unaffected.

**Conclusion:**

Short‐term incubation in serum‐free Accell medium restores MRGPRX2 expression and signaling in cultured skin MCs without impairing viability. This simple adjustment yields a practical and reliable model for MRGPRX2‐focused studies.

To the Editor

MRGPRX2 (Mas‐related G protein‐coupled receptor member X2) has attracted substantial attention on account of its selective expression in mast cells (MCs), the existence of hundreds of ligands (both exogenous and endogenous) and its potency as elicitor of degranulation, linking the receptor to skin diseases associated with MC activation like urticaria, atopic dermatitis, prurigo, and rosacea, for which the stimulatory path involved remained unknown for decades [1, 2]. Accordingly, pharmaceutical companies have embarked on the development of MRGPRX2‐targeting antagonists. Their testing requires the appropriate in vitro/ex vivo systems. The MC line LAD2 is widely used owing to its robust responsiveness to MRGPRX2 agonists. However, LAD2 cells differ from skin MCs regarding degranulation, receptor internalization and desensitization, partially attributable to perturbations in their *β*‐arrestin system [3, 4]. These findings emphasize the indispensability of skin MCs in both mechanistic studies and translational applications of drug development.

While dermal MCs represent the gold standard in MRGPRX2 in vitro research, the cells reduce MRGPRX2 expression during culture as a result of the high levels of stem cell factor (SCF) and (to a lesser degree) interleukin‐4 (IL‐4), which are required for MC survival and proliferation, but elicit downregulation of MRGPRX2 [5]. Short term removal of cytokines can reestablish MRGPRX2 to a certain degree [5], but the absence of SCF blunts viability and attenuates metabolism, making growth factor withdrawal to boost MRGPRX2 impractical. However, culture‐expanded skin MCs, available in relatively large numbers, maintain many attributes imparted by the skin environment and are an attractive system to study MRGPRX2 functionality [6].

We noticed that skin MCs kept in Accell medium (used for RNA interference) consistently express higher levels of MRGPRX2 and display enhanced degranulation toward its ligands.

Aiming to reinstate MRGPRX2 expression in these otherwise ideally suited cells, we set out to systematically investigate this phenomenon.

Besides SCF, skin MCs require fetal calf serum (FCS) for growth, which is likewise a negative regulator of MRGPRX2. Accell medium is FCS‐free, and it maintains skin MC survival better than other media (provided SCF is present), though it does not support mitogenesis.

To find an optimized system for MRGPRX2 studies, we compared the impact of medium on MRGPRX2 expression and functional outputs. MCs were isolated from foreskin and cultured in standard medium (basal Iscove, 10% FCS, SCF + IL‐4) for 2.5–4 weeks. For experiments, cells were transferred to Accell/100 ng/mL SCF or fresh standard medium (10% FCS, 100 ng/mL SCF). After 3 days, MRGPRX2 expression was measured by flow‐cytometry; cells were stimulated with codeine or Substance P and degranulation was assessed by *β*‐hexosaminidase, tryptase, and histamine release. Western blots served to study signal transduction. All Methods are described in the online supplement.

MRGPRX2 was upregulated in Accell vis‐à‐vis standard medium to 172.0 ± 15.4% of control (Figure 1a,b). MRGPRX2 expression correlates with MRGPRX2‐triggered exocytosis [5]. Also in the current study, the heightened MRGPRX2 surface density gave rise to enhanced degranulation, as determined by *β*‐hexosaminidase release (Figure 1c,d). Similarly, Accell‐cultured MCs released more tryptase than those in standard medium, further supporting their heightened secretory capability (Figure 1e,f). The same trend was also observed with regard to histamine secretion (Figure 1g,h); thus, the three mediators yielded congruent results. Variations in Figure 1 stem from the fact that each experimental point represents MCs from a different donor pool. This is in accordance with previously published evidence showing a high degree of interindividual variability regarding MRGPRX2 biology for example, reference [7]. FcεRI‐mediated functions did not suffer major changes in Accell medium, as determined for tryptase and *β*‐hexosaminidase (Supporting Information S1: Figure S1a,b). Therefore, these cells seem to be useful for studies of the IgE receptor, allowing simultaneous investigation of allergic and pseudo‐allergic/neurogenic activation programs. We note, however, that our current data only demonstrate the presence of functional FcεRI stimulated by one crosslinking strategy. Importantly, FcεRI function is enhanced in standard MC cultures compared with ex vivo cells, so conditions to further augment FcεRI‐dependent responses are less necessary [5]. Finally, we addressed the impact of medium on signaling events elicited via MRGPRX2. Receptor ligation leads to rapid ERK and Akt phosphorylation in skin MCs, while other modules (including JNK, p38 and NFκB) are less strongly (or not) activated [8]. Indeed, MRGPRX2 signaling was more efficiently induced in Accell‐preincubated cells compared to those kept in standard medium. Both pERK and pAKT signals were stronger and of prolonged duration (Figure 1i). Notably, the PI3K/AKT module strongly contributes to MRGPRX2‐elicited degranulation of skin MCs [8]. Therefore, a short‐time switch from FCS‐containing standard medium (required for optimal skin MC growth) to serum‐free Accell efficiently upregulates MRGPRX2 expression and function to increase the cells' utility in MRGPRX2 directed research.

**FIGURE 1:**
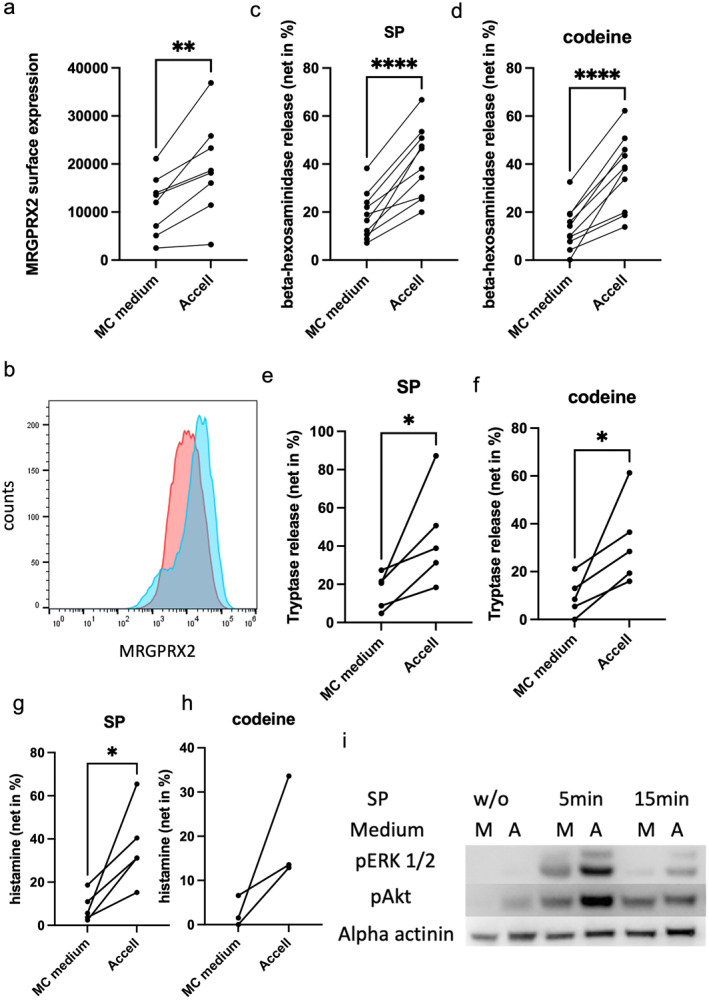
MRGPRX2 expression and function are upregulated in Accell medium. Skin MCs were cultured in standard medium or Accell medium for 3 days, as described in Supporting Information S1: Supplementary Methods. (a, b) Surface MRGPRX2 expression was measured by flow cytometry. (a) Cumulative results of 8 separate experiments (mean fluorescence intensity). (b) Representative histograms: standard medium (red), or Accell (blue). (c–h) Mast cells (MCs) were stimulated by (c, e, g) Substance P (SP, 30 μM) or (d, f, h) codeine (100 μg/mL) for 60 min. Degranulation was determined by (c, d) *β*‐hexosaminidase or (e, f) tryptase, and (g, h) histamine release and is given as net release in % of the total mediator contents; dots of the same MC cultures are interconnected, *n* = 3–10. (i) Phosphorylation of key kinases in skin MCs elicited by SP for different times. A: Accell medium, M: standard MC medium. One representative membrane (out of two) is shown. *p < 0.05, **p < 0.01, ****p < 0.0001. Each experimental point corresponds to MCs from one donor pool (each encompassing 2–14 donors).

Microenvironmental cues critically impact on the MRGPRX2 receptor system. In accordance, studies using CD34+ derived MCs and other cell types likewise observed that the culture medium has a decisive impact on MRGPRX2 function [9]. There is a pressing need for more accessible in vitro models that closely mimic native MC behavior and are available in sufficient quantities for screening applications and mechanistic studies. We have identified conditions to reestablish MRGPRX2 in skin‐derived MCs even in the presence of SCF, thereby offering a tool for translational research. Culture‐expanded skin MCs are available in larger quantities than ex vivo skin MCs (expansion by ˜ 8‐fold), while still sharing many properties with the latter [6]. Our optimized culture approach may thus help provide answers to unresolved questions surrounding MRGPRX2 pathophysiology.

## Author Contributions

**Zhuoran Li:** conceptualization, data curation, formal analysis, investigation, methodology, writing – review and editing. **Jean Schneikert:** writing – review and editing, data curation, investigation. **Anja Wegner:** investigation, writing – review and editing. **Torsten Zuberbier:** resources, supervision, funding acquisition, writing – review and editing. **Magda Babina:** conceptualization, formal analysis, writing – review and editing, methodology, writing–original draft, supervision.

## Consent

Donor skins, which otherwise would be disposed of, were obtained from circumcisions (foreskin) with written informed consent of the patients or their legal guardians.

## Conflicts of Interest

Magda Babina has received consulting fees or research funding from Septerna, Escient, Health Advances, Evoimmune, Deep Apple, Alector, and Incyte. Torsten Zuberbier has received honoraria for lectures from Amgen, AstraZeneca, AbbVie, ALK–Abelló, Almirall, Astellas, Bayer Health Care, Bencard, Berlin Chemie, FAES Farma, HAL Allergie GmbH, Henkel, Kryolan, Leti, L'Oreal, Meda, Menarini, Merck Sharp & Dohme, Novartis, Nuocor, Pfizer, Sanofi, Stallergenes, Takeda, Teva, UCB, and Uriach; Fees for industry consulting were received from Abivax, Almirall, Bluprint, Celldex, Celltrion, Novartis, and Sanofi; in addition he declares non‐paid organizational affiliations: Committee member, “Allergic Rhinitis and its Impact on Asthma” (ARIA), Member of the Board, German Society for Allergy and Clinical Immunology (DGAKI), Head, European Centre for Allergy Research Foundation (ECARF), President, Global Allergy and Asthma Excellence Network (GA^2^LEN), and Member, Committee on Allergy Diagnosis and Molecular Allergology, World Allergy Organisation (WAO). The other authors declare no potential conflict of interest.

## Supporting information


Supporting Information S1

## Data Availability

No datasets were generated or analyzed during the current study.

## References

[1] M. Kumar , K. Duraisamy , and B. K. Chow , “Unlocking the Non‐IgE‐Mediated Pseudo‐Allergic Reaction Puzzle With Mas‐Related G‐Protein Coupled Receptor Member X2 (MRGPRX2),” Cells 10, no. 5 (2021): 1033, 10.3390/cells10051033. 33925682 PMC8146469

[2] H. Kühn , P. Kolkhir , M. Babina , et al., “Mas‐Related G Protein‐Coupled Receptor X2 and Its Activators in Dermatologic Allergies,” Journal of Allergy and Clinical Immunology 147, no. 2 (2021): 456–469, 10.1016/j.jaci.2020.08.027. 33071069

[3] Z. Li , J. Schneikert , G. Bal , et al., “Intrinsic Regulatory Mechanisms Protect Human Skin Mast Cells From Excessive MRGPRX2 Activation: Paucity in LAD2 (Laboratory of Allergic Diseases 2) Cells Contributes to Hyperresponsiveness of the Mast Cell Line,” Journal of Investigative Dermatology 145, no. 5 (2025): 1215–1219.e4, 10.1016/j.jid.2024.10.593. 39481529

[4] Z. Li , J. Schneikert , G. Bal , T. Zuberbier , and M. Babina , “Icatibant Acts as a Balanced Ligand of MRGPRX2 in Human Skin Mast Cells,” Biomolecules 15, no. 9 (2025): 1224, 10.3390/biom15091224. 41008531 PMC12466958

[5] M. Babina , Z. Wang , M. Artuc , S. Guhl , and T. Zuberbier , “MRGPRX2 Is Negatively Targeted by SCF and IL‐4 to Diminish Pseudo‐Allergic Stimulation of Skin Mast Cells in Culture,” Experimental Dermatology 27, no. 11 (2018): 1298–1303, 10.1111/exd.13762. 30091263

[6] S. Akula , S. R. Tripathi , K. Franke , S. Wernersson , M. Babina , and L. Hellman , “Cultures of Human Skin Mast Cells, an Attractive In Vitro Model for Studies of Human Mast Cell Biology,” Cells 13, no. 1 (2024): 98, 10.3390/cells13010098. 38201301 PMC10778182

[7] M. Babina , S. Guhl , M. Artuc , and T. Zuberbier , “Allergic FcεRI‐ and Pseudo‐Allergic MRGPRX2‐Triggered Mast Cell Activation Routes Are Independent and Inversely Regulated by SCF,” Allergy 73, no. 1 (2018): 256–260, 10.1111/all.13301. 28859248

[8] Z. Wang , K. Franke , G. Bal , Z. Li , T. Zuberbier , and M. Babina , “MRGPRX2‐Mediated Degranulation of Human Skin Mast Cells Requires the Operation of G_αi_, G_αq_, Ca++ Channels, ERK1/2 and PI3K‐Interconnection Between Early and Late Signaling,” Cells 11, no. 6 (2022): 953, 10.3390/cells11060953. 35326404 PMC8946553

[9] A. Toscano , J. Elst , A. L. Van Gasse , et al., “Mas‐Related G Protein‐Coupled Receptor MRGPRX2 in Human Basophils: Expression and Functional Studies,” Frontiers in Immunology 13 (2022): 1026304, 10.3389/fimmu.2022.1026304. 36726977 PMC9885256

