# Supplementary material for: A Novel Cell Culture System to Improve MRGPRX2 Research in Human Skin Mast Cells
Source: Clin Transl Allergy. 2025 Oct 16;15(10):e70112. doi: 10.1002/clt2.70112 (PMC12529870; doi:10.1002/clt2.70112)
Supplement: Supplementary file 1 — Supporting Information S1 [file CLT2-15-e70112-s001.docx]

**Supplementary Material**

**Supplementary Figure**

**FcεRI function is not compromised in Accell® medium**

Skin MCs were cultured in standard medium or Accell® medium for 3 days and then stimulated by FcεRI-crosslinking (CL) as described in Suppl. Methods. Degranulation was determined by (a) tryptase or (b) β-hexosaminidase secretion and is given as net release in % of the total mediator contents; dots of the same MC cultures are interconnected, n=5-7. * P < 0.05.

**Methods**

**SkMC purification, culture, and stimulation**

This was an unlinked anonymous study. Donor skins, which otherwise would be disposed of, were obtained from circumcisions (foreskin) with written informed consent of the patients or their legal guardians, as in previous studies from our group [1-3]. The study was approved by the Ethics Committee of the Charité Universitätsmedizin Berlin (EA1/204/10) and experiments were conducted according to the Declaration of Helsinki Principles. MCs were isolated as described [4, 5]. Briefly, skin samples (foreskins pooled from 2–14 donors) were cut into strips and treated with dispase at 3.5 U/ml (BD Biosciences, Heidelberg, Germany) at 4 °C overnight. The epidermis was removed; the dermis was chopped to homogeneity and digested with an enzyme cocktail containing 1.5 mg/ml of collagenase type 1 (Worthington, Lakewood, NJ, USA), 0.75 mg/ml hyaluronidase type 1-S (Sigma, Steinheim, Germany), and DNase I at 10 µg/ml (Roche, Basel, Switzerland) at 37 °C in a shaking water bath for 75 min. The cells were separated from the remaining tissue by filtration and incubated with anti-human c-Kit microbeads for positive selection by the Auto-MACS (both from Miltenyi Biotec, Bergisch Gladbach, Germany). MC purity (>98%) was assessed by toluidine blue staining and viability (>99%) by trypan blue. MCs isolated from foreskin were cultured in standard medium (basal Iscove, 10% FCS, SCF at 100 ng/ml and IL-4 at 10 ng/ml) for 2.5-4 weeks. For experiments, cells were transferred to Accell® medium (100 ng/ml SCF) or to fresh standard medium (containing 10% FCS, 100 ng/ml SCF) and used after 3 days.

**β-hexosaminidase release assay**

Detection of β-hexosaminidase was performed as described [2, 5]. Briefly, cells were treated with vehicle (spontaneous release), or challenged with codeine-phosphate at 100 μg/ml (solution prepared by the Charité pharmacy at 0.9% in water), substance P (SP) at 30 μM (Bachem, Budendorf, Switzerland), or the functional anti-FcεRIα antibody AER-37 (eBioscience, San Diego, CA, USA) at 0.2 µg/ml for 60 min in PAG-CM buffer (250 mM Pipes, 1.19M NaCl, 50 mM KCl, 28 mM CaCl_2_, 14 mM MgCl_2_, 0.1% glucose, 0.001% human serum albumin, pH7.4). Supernatants (SNs) were collected, and the pelleted MCs were rapidly frozen with 100 μl H_2_O at −80°C. After thawing, 50 μl of SNs and lysates were incubated with 50 µl of 4-methyl umbelliferyl-N-acetyl-beta-D-glucosaminide (Sigma-Aldrich, Munich, Germany) solution at 5 μM in citrate buffer (100 mM, pH 4.5) and incubated for 60 min at 37°C. 100µl of a 100 mM sodium carbonate buffer (pH 10.7) was added to stop the reaction. Fluorescence intensity at emission wavelength of 460 nm after excitation at 355 nm was determined on the VICTOR X5 2030 Multilabel HTS Microplate Reader (Perkin Elmer, Berlin, Germany). Percent β-hexosaminidase release = [fluorescence intensity SN / (fluorescence intensity SN + fluorescence intensity lysate)] × 100. Net release was calculated by subtracting spontaneous release.

**Tryptase assay**

Tryptase activity was measured according to an established protocol [4, 6-8]. 50,000 skMCs/100 µl in PAG-CM buffer were exposed to the given stimuli for 1 h, as described above. The plate was spun and 50 µl of the supernatant used for quantification. For complete tryptase content, skMCs were lysed in water at 50,000/100 µl followed by rapid freezing at -80 °C. 50 µl samples thereof were diluted (typically 1:10) and analyzed at least in triplicate. Enzyme activity was determined by monitoring the cleavage of the peptide N-CBZ-Gly-Pro-Arg-pNA (Sigma-Aldrich, Munich, Germany) at 0.5 mg/ml. To 50 µl of each sample, 150 µl of sample buffer (150 mM Tris pH 7.6, 300 mM KCl, 50 µg/ml heparin) were added. To eliminate confounding enzyme activities, alpha-1 antitrypsin at a final concentration of 1 mg/ml served to suppress trypsin-like proteases. The changes in optical density per minute, caused by the cleavage of the substrate, were monitored, and recorded by measuring absorbance at 405 nm every 2 minutes on the VICTOR X5 2030 Multilabel HTS Microplate Reader. Tryptase concentration in the samples was calculated relative to a standard curve prepared with Tryptase beta-2 Recombinant Protein (ProSci Inc. #91-456, Fort Collins, CO, USA) diluted in 8 two-fold dilution steps from 1000 ng/ml down to 7.8125 ng/ml.

**Histamine release assay**

Histamine was measured by using the HTRF (Homogenous Time-Resolved Fluorescence) Histamine Dynamic Detection Kit from Revvity (catalog number 62HTMDPEG, Hamburg, Germany) according to the manufacturer’s instructions. Briefly, an anti-histamine donor antibody labelled with a fluorescent dye (Europium cryptate) is excited with a light source. Fluorescence Resonance Energy Transfer (FRET) occurs when the antibody is in close proximity of a histamine acceptor reagent also labelled with a fluorescent dye (d2) that in turn emits at 665nm. Histamine present in the sample competes for binding to the antibody and thereby prevents FRET from occurring. Typically, 50,000 skMCs/100 µl in PAG-CM buffer (see above) were exposed to the given stimuli for 1 h, as described above. The plate was spun and 7 µl of each supernatant diluted 1:10 used for quantification. For complete histamine content, skMCs were lysed in water at 50,000/100 µl followed by rapid freezing at -80 °C. 50 µl samples thereof were diluted (typically 1:100) and 7 µl analysed. For the assay, 7 µl of sample were first mixed with 4 µl of acylation buffer in a 96 micro-well plate, then with 2 µl of acylation reagent. After incubation at room temperature for 15 minutes, 4 µl of d2 reagent were added, followed by 4 µl of anti-histamine Eu Cryptate antibody. The plate was incubated at room temperature for one hour and the emitted signals at 665 nm and 620 nm were recorded by the VICTOR X5 2030 Multilabel HTS Microplate Reader. The ratios of the acceptor (665 nm) and donor (620 nm) emission signals were calculated and compared to a standard curve obtained with known concentrations of histamine. Results were expressed as the net percentage of released histamine after subtracting the spontaneous release without inducer of degranulation.

**Immunoblot analysis**

Detection of Akt and ERK1/2 phosphorylation was performed as described [5]. SkMCs were stimulated (at 5×10^5^ /ml in serum-free medium) by SP 30 μM for 5 min or 15 min. After centrifugation, cells were boiled in an SDS-PAGE sample buffer for 10 minutes. Lysates were separated through 4-12% Bis-Tris gels (Thermo Fisher Scientific, Berlin, Germany). After transferring to a membrane and incubation with antibodies, proteins were visualized by a chemiluminescence assay (Weststar Ultra 2.0, Cyanagen, Bologna, Italy), and bands were recorded on a chemiluminescence imager (Fusion FX7 Spectra, Vilber Lourmat, Eberhardzell, Germany). The following primary antibodies, all purchased from Cell Signaling Technology (Frankfurt am Main, Germany), were used: anti-phosphorylated ERK1/2 (1:1000 dilution, T202/Y204, #9101), anti-phosphorylated Akt (1:1000 dilution, Ser473, #9271), anti-α-actinin (1:1000 dilution, #6487). Goat anti-rabbit IgG peroxidase-conjugated antibody was administered as the detection antibody (1:10,000 dilution, Merck, Darmstadt, Germany; #AP132P).

**Flow cytometry**

Flow cytometry was carried out as described [9]. MCs were blocked with human AB serum (Biotest, Dreieich, Germany) for 15 minutes at 4 °C and then stained with anti-MRGPRX2-APC antibody (Biolegend, #359006, Amsterdam, Netherlands) for 30 minutes at 4 °C.  Corresponding isotype controls were used in each experiment. After incubation, cells were washed in phosphate-buffered saline (PBS) and resuspended in fluorescence activated cell sorting (FACS) buffer consisting of 2 % fetal bovine serum in PBS. The cells were immediately processed on a Sony ID7000™ Spectral Cell Analyzer (Berlin, Germany) and gated on the population of identifiable, healthy cells in the forward scatter/side scatter plot, excluding debris and dead cells. The data were analyzed with the FlowJo analysis software (FlowJo LLC, Ashland, OR, USA).

**References**

1. Babina, M., et al., *Allergic FcεRI- and pseudo-allergic MRGPRX2-triggered mast cell activation routes are independent and inversely regulated by SCF.* Allergy, 2018. **73**(1): p. 256-260.

2. Wang, Z., et al., *IL-33 and MRGPRX2-Triggered Activation of Human Skin Mast Cells-Elimination of Receptor Expression on Chronic Exposure, but Reinforced Degranulation on Acute Priming.* Cells, 2019. **8**(4).

3. Wang, Z., et al., *Cytokine Stimulation by MRGPRX2 Occurs with Lower Potency than by FcεRI Aggregation but with Similar Dependence on the Extracellular Signal-Regulated Kinase 1/2 Module in Human Skin Mast Cells.* J Invest Dermatol, 2022. **142**(2): p. 414-424.e8.

4. Babina, M., et al., *Phenotypic variability in human skin mast cells.* Exp Dermatol, 2016. **25**(6): p. 434-9.

5. Babina, M., et al., *Yin-Yang of IL-33 in Human Skin Mast Cells: Reduced Degranulation, but Augmented Histamine Synthesis through p38 Activation.* J Invest Dermatol, 2019. **139**(7): p. 1516-1525.e3.

6. Guhl, S., et al., *Mast cell lines HMC-1 and LAD2 in comparison with mature human skin mast cells--drastically reduced levels of tryptase and chymase in mast cell lines.* Exp Dermatol, 2010. **19**(9): p. 845-7.

7. Guhl, S., et al., *Skin mast cells develop non-synchronized changes in typical lineage characteristics upon culture.* Exp Dermatol, 2014. **23**(12): p. 933-5.

8. Harvima, I.T., et al., *Biochemical and histochemical evaluation of tryptase in various human tissues.* Arch Dermatol Res, 1989. **281**(4): p. 231-7.

9. Babina, M., et al., *MRGPRX2 Is the Codeine Receptor of Human Skin Mast Cells: Desensitization through β-Arrestin and Lack of Correlation with the FcεRI Pathway.* J Invest Dermatol, 2021. **141**(5): p. 1286-1296.e4.
